# Effects of Dynamic Resilience on the Reactivity of Vagally Mediated Heart Rate Variability

**DOI:** 10.3389/fpsyg.2020.579210

**Published:** 2021-01-20

**Authors:** Luke Crameri, Imali T. Hettiarachchi, Samer Hanoun

**Affiliations:** Institute for Intelligent Systems Research and Innovation, Deakin University, Geelong, VIC, Australia

**Keywords:** heart rate variability, dynamic resilience, cognitive assessment, physiological markers, human performance psychology

## Abstract

Dynamic resilience is a novel concept that aims to quantify how individuals are coping while operating in dynamic and complex task environments. A recently developed dynamic resilience measure, derived through autoregressive modeling, offers an avenue toward dynamic resilience classification that may yield valuable information about working personnel for industries such as defense and elite sport. However, this measure classifies dynamic resilience based upon in-task performance rather than self-regulating cognitive structures; thereby, lacking any supported self-regulating cognitive links to the dynamic resilience framework. Vagally mediated heart rate variability (vmHRV) parameters are potential physiological measures that may offer an opportunity to link self-regulating cognitive structures to dynamic resilience given their supported connection to the self-regulation of stress. This study examines if dynamic resilience classifications reveal significant differences in vagal reactivity between higher, moderate and lower dynamic resilience groups, as participants engage in a dynamic, decision-making task. An amended Three Rs paradigm was implemented that examined vagal reactivity across six concurrent vmHRV reactivity segments consisting of lower and higher task load. Overall, the results supported significant differences between higher and moderate dynamic resilience groups' vagal reactivity but rejected significant differences between the lower dynamic resilience group. Additionally, differences in vagal reactivity across vmHRV reactivity segments within an amended Three Rs paradigm were partially supported. Together, these findings offer support toward linking dynamic resilience to temporal self-regulating cognitive structures that play a role in mediating physiological adaptations during task engagement.

## 1. Introduction

Resilience profiling is common practice when recruiting for occupations working within dynamic operational environments; for example, elite sporting and Defense domains (Park et al., 2017; Hill et al., 2018a). While theoretically divisive, resilience is often conceived as a stable trait that promotes one's ability to adapt positively in the face of adversity (Rutter, 2006; Cicchetti, 2010; Southwick et al., 2014; Vella and Pai, 2019). However, discussions targeting the construct value of trait resilience in the human performance domain have challenged its applicability; as its causal chain approach (i.e., a causal variable that determines behavioral outcomes) fails to capture the dynamic process of resilience under load (Hill et al., 2018a). This inspired the conception of the *dynamic resilience* theoretical framework, in which its proposed methods aim to capture and profile the process of resilience as it unfolds (Hill et al., 2018b; Crameri et al., 2020b). While this has currently been achieved by tracking objective performance outputs, previous research has called for more holistic methods toward profiling dynamic resilience that comprises examining psychophysiological relationships between dynamic resilience and physiological adaptations to in-task stress (Crameri et al., 2020a,b). In particular, whether attributes of dynamic resilience mediate cardiac vagal activity during task stress (Crameri et al., 2020a).

Traditionally, previous research directed at profiling resilience in human performance areas predominantly focused on personality traits and health initiatives that mitigate susceptibility to psychopathological effects of stress (Bartone, 1999; Bowles et al., 2010). Resilience, and other construct-related concepts (e.g., hardiness and grit), are quantified through latent psychometric self-reporting measures that enquired about protective factors (e.g., health, social-outlets, stress perceptions) believed to mediate one's overall resilience (Bartone, 1999; Windle et al., 2011). From a health perspective, resilience measures, such as the popular Connor-Davidson Resilience Scale (Connor and Davidson, 2003), have demonstrated value in their application; as inverse relationships have been empirically supported between trait resilience and susceptibility to psychopathological outcomes (Nezhad and Besharat, 2010). Susceptibility to psychopathological outcomes is linked to poor self-regulation (i.e., emotional-regulation and attentional-regulation), in which the individual may become over aroused and unable to cope with in-coming stressors (Koenig, 2020; Langer et al., 2020). Hence, characteristics of self-regulation have been examined to evaluate if it is mediated by one's trait resilience (Hu et al., 2015; Armstrong et al., 2018). Across various domain, empirical findings suggest that those possessing higher trait resilience often exhibit more efficient self-regulation tendencies toward stressors than those possessing lower trait resilience (Souza et al., 2013; Hildebrandt et al., 2016; Lü et al., 2016; Hourani et al., 2020; Perna et al., 2020).

As trait resilience measures do not capture the temporal process of resilience as it unfolds, it fails to evaluate in-situation coping (Hill et al., 2018a,b). Instead, the link between trait resilience and performance is a global inference that predicts future long-term success (Bryan et al., 2019). This is evident in research demonstrating that higher trait resilience enhances the likelihood of completing military training, academic pursuits, and achieving athletic success (Nezhad and Besharat, 2010; Skomorovsky and Stevens, 2013; Eskreis-Winkler et al., 2014). While valuable for recruitment purposes, it does not account for acute changes in psychological statuses due to temporal factors such as burnout effects, and environmental and social factors (Hill et al., 2018a). Furthermore, trait resilience measures are administered via subjective self-reporting methods. This leaves these measures susceptible to bias responses, as individuals may manipulate resilience profiling due to intrinsic motivation and peer-pressures (Walker et al., 2017). To overcome these limitations, Hill et al. (2018a,b) offered an alternative resilience framework that sought to assess resilience through more objective, in-situational methods. Titled dynamic resilience, Hill et al. (2018a) defined it as “the dynamic process by which a biopsychosocial system returns to the previous level of functioning, following a perturbation caused by a stressor” (p. 367). Its conceptual framework adopts dynamic system principles in which resilience is conceived as a malleable dynamic process built upon the complex interactions between protective, environmental, and task factors over time. Together, these factors interact with the history of the task and generate psychological momentum that enhance or diminish one's dynamic resilience to cope with in-situational task stress.

Guidelines for dynamic resilience measurement offered potential progress through first-process autoregressive [AR(1)] modeling (Hill et al., 2018a). Based upon the critical slowing down literature (Dai et al., 2012; Dakos et al., 2012; van de Leemput et al., 2014), it is postulated that tracking behavior across a time series, via AR(1) modeling, may reveal temporal aspects of individuals' recovering from task induced load and stress. This could potentially lead to the identification of performance “tipping point” in which individuals' can no longer sustain optimal task performance (Hill et al., 2018b). Research evaluating the feasibility of AR(1) modeling has empirically supported its application for dynamic resilience measurement (Crameri et al., 2020b). More specifically, AR(1) modeling of individuals' task performance facilitated the tracking of individuals' dynamic resilience, in which dynamic resilience could be quantified and further classified by examining the autocorrelational patterns across higher and lower load sections of a task. The groups appeared to represent the phasic decline of group members' dynamic resilience over the task time period. Thereby, demonstrating a potentially sensitive in-situation measure of dynamic resilience for human performance profiling. However, while potentially useful for human performance domains, the dynamic resilience measure does not present support for cognitive structures typically associated with trait resilience. Instead, the measure may be more reflective of temporal task proficiency as it is derived from task performance. Given this, it is still unclear if cognitive structures related to the self-regulation of stress is associated with dynamic resilience.

### 1.1. Physiological Manifestations of Stress

The occurrence of stress activates complex neural activity purposed with orienteering the human toward adaptive responses (Charmandari et al., 2005). The primary neural systems responsible for the response to stress include the prefrontal cortex, the hippocampus and amygdala of the limbic system, and the hypothalamus (Baumann and Turpin, 2010). Together, these neural systems, along with the spinal cord, make up part of the central nervous system (CNS) that sends signals to the peripheral nervous system to promote allostasis within the human (Kirschbaum et al., 1999; Charmandari et al., 2005; McEwen, 2005; Stephens and Wand, 2012). The peripheral nervous system reflects the nerves and ganglia throughout the body, and facilitates the process of relaying messages from the CNS to the body. Upon the occurrence of a stressor, the peripheral nervous system activates the functioning of the autonomic nervous system and the hypothalamic–pituitary–adrenal (HPA) axis of the endocrine system (Kirschbaum et al., 1999; Charmandari et al., 2005; McEwen, 2005; Stephens and Wand, 2012). These systems are interconnected and activated involuntarily, with each system comprising distinct functions toward a coordinate adaptive response to stress (Rotenberg and McGrath, 2016). The ANS acts as the first-responder to stress through quick physiological adaptations that prepares the human for adaptive responses to potential cognitive and environmental challenges or threats (Tsigos and Chrousos, 2002; Seery, 2011). The ANS regulates physiological responses to stress via two subsystems, the sympathetic nervous system (SNS) and the parasympathetic nervous system (PNS) (de Looff et al., 2018).

Early beliefs of the SNS and PNS relationship were viewed as antagonistic, in which the SNS would act as the excitatory and the PNS as the inhibitory function of the ANS (Moses et al., 2007). This view was founded upon the understanding of the role that the SNS plays during fight/flight responses; in which SNS activity generates physiological adaptations such as increases in heart rate (HR), dilation of pupils and metabolic rate, while decreasing digestive and urinary functions (Martini et al., 2018). Meanwhile, the PNS inhibits SNS activity during *rest and digest* situations, in which visceral activity is stimulated and the physical adaptations of the SNS are reversed (e.g., decreases in HR and increases in digestive functioning) (Kim et al., 2018). More recent advancements in neuroanatomy and physiology have demonstrated that the antagonistic view of the SNS and PNS is oversimplified, and that the relationship between the subsystems are far more complex (Herring et al., 2019; Benarroch, 2020). While some physiological adaptations arise via an excitatory/inhibitory relationship between the SNS and PNS, other physiological adaptations are mediated via collaborative stimulation of both the SNS and PNS, or independently through just one of the subsystems (Martini et al., 2018; Benarroch, 2020). To this end, the subsystems facilitate the ANS to adaptively respond to stress and return to physiological homeostasis once the source of the stress has been resolved (Herring et al., 2019; Tobaldini et al., 2020).

Profiling the efficiency of an individual's ANS has offered invaluable insights into self-regulation when enduring various types of stressors (Hjortskov et al., 2004; McDuff et al., 2014; An et al., 2020; Perna et al., 2020). To achieve this, the PNS is predominantly examined over the SNS as it plays a primary role in regulating physiological adaptations when facing both physical and psychological stressors (Thayer et al., 2009). PNS activity can be indexed through cardiac vagal tone, which reflects the interaction between the PNS and the vagus nerve (Porges, 1995). The vagus nerve serves an important purpose for self-regulation as it provides the functional and structural link connecting the brain to the heart (Laborde et al., 2017). Utilizing this connection, the PNS stimulates the vagus nerve to release acetylcholine neurotransmitters that promote tonic inhibitory control of the heart in efforts to sustain homeostatic levels (Thayer et al., 2009; Kim et al., 2018). The efficiency of this process is often derived through the measurement of vagally-mediated HRV (vmHRV) parameters in which higher levels of vmHRV parameters are linked to superior self-regulation and cognitive performance outcomes (Malik et al., 1996; Berntson et al., 1997; Appelhans and Luecken, 2006; Thayer et al., 2009; Pendleton et al., 2016; Shaffer and Ginsberg, 2017).

Theoretically, the neurovisceral integration (NVI) model offers a functional framework connecting vmHRV, self-regulation and cognitive performance (Thayer and Lane, 2000; Thayer et al., 2009; Laborde et al., 2017; Smith et al., 2017). In short (see Thayer et al., 2009; Smith et al., 2017 for detailed descriptions), the NVI model is founded upon the role of the central autonomic network (CAN); a component of the CNS that comprises different brain structures within the prefrontal cortex and exerts control over internal-regulation (Laborde et al., 2018). The CAN integrates visceralmotor, neuroendocrine, and behavior response with emotion, attention, and cognitive executive functioning for goal-directed behaviors and adaptability (Benarroch, 1993; Thayer and Lane, 2000; Smith et al., 2020). This is facilitated through vagal feedback loops, that provides an anatomical pathway connecting the CNS and the ANS (Koenig, 2020). The afferent vagal loop facilitates the retrieval of status updates regarding sensory organ functioning and environmental demands to the CAN, while the efferent vagal loop enable the CAN to respond and innervate organs to meet demands. The CAN output delivered via the efferent vagal loop is predominantly mediated by preganglionic sympathetic and parasympathetic neurons (Thayer et al., 2009). The interaction of these neurons with the sino-atrial node of the heart creates the variable cardiac rhythm, linking the CAN output directly with HRV. As the CNS receives a high quantity of afferent information from sensory organs and the environment, the NVI model postulates that vmHRV reflects the functioning status of the CNS-ANS integration (Smith et al., 2017). In particular, cardiac vagal control, indexed by vmHRV, is likely reflective of inhibitory control processes within the prefrontal cortex that promote self-regulation and cognitive performance (Thayer and Lane, 2000; Smith et al., 2017).

vmHRV measures are derived from time domain and frequency domain analyses (Laborde et al., 2017; Shaffer and Ginsberg, 2017). Early guidelines for HRV parameters offered various directions toward standardizing practices for HRV application and interpretability (Malik et al., 1996; Berntson et al., 1997). However, more recent reviews of the HRV literature have highlighted the inconsistencies of various HRV parameters across research; ultimately disputing the validity of some HRV parameters as indexes of sympathetic and parasympathetic activity (Hayano and Yuda, 2019). Of the most common and well-received vmHRV parameters to index PNS activity comprises the root mean square of successive differences between normal heartbeats (rMSSD) and the percentage of adjacent NN intervals that differ from each other by more than 50 ms (pNN50) of the time domain and the high frequency band (HF-HRV) of the frequency domain (Malik et al., 1996; Berntson et al., 1997; Laborde et al., 2017). The three HRV parameters are correlated, with the difference being that HF-HRV is highly influenced by individuals' respiratory cycles and that rMSSD and pNN50 are associated with the short-term, rapid changes in HR (Stein et al., 1994; DeGiorgio et al., 2010; Shaffer et al., 2014; Shaffer and Ginsberg, 2017). Together, these three vmHRV parameters are widely implemented within psychophysiological research examining the effects of resilience on the self-regulation of stress (Thompson et al., 2015; Spangler et al., 2018; Flatt et al., 2020).

Vagal reactivity studies detail an approach toward profiling individual's vagal response to stress (Laborde et al., 2018). Vagal reactivity refers to the process in which the PNS withdraws cardiac vagal control during encounters with stressors (Jentsch and Wolf, 2020). In other words, it reflects the phasic vagal changes that occur from rest to stress. Laborde et al. (2017) proposed a “*Three Rs: Rest, Reactivity and Recovery*” experimental paradigm to profile the cardiac vagal reactivity process. This experimental paradigm comprises the measurement of vmHRV during three phases: a resting baseline phase, a challenging or stressful phase, and a resting recovery phase (Laborde et al., 2018). Cardiac vagal reactivity is evaluated via the phasic differences in cardiac vagal control withdrawal, indexed by vmHRV. Small decreases or increases in vmHRV during the reactivity phase and the recovery phase indicates greater self-regulation, whereas moderate to large decreases in vmHRV reflects poorer self-regulation. However, as Hottenrott et al. (2019) points out in their commentary of the Three Rs paradigm, this experimental design applies to single global events (i.e., rest, task, recovery), and not the temporal components within the task. Hence, to align the Three Rs paradigm with the dynamic resilience framework, amendments to the rest, reactivity, recovery segments will be required; with an emphasis on capturing how individuals physiologically react to sudden difficulties and setbacks as they progress through a task.

To summarize, dynamic resilience presents a relatively new model of resilience that may hold valued sensitivity in human performance evaluation. However, while Crameri et al. (2020b)'s dynamic resilience measure has revealed sensitivity in classifying individuals into dynamic resilience groups based on in-task performance, further research needs to be conducted to identify if dynamic resilience characteristics links to self-regulating cognitive structures that play a role in mediating physiological adaptations during task engagement. To address this, this paper aims to evaluate the application of the dynamic resilience measure on vmHRV. This is due to the proposed link vmHRV offers toward indexing emotional-regulation and attentional-regulation via cardiac vagal control (Thayer et al., 2009). Achieving this goal will be sought through the evaluation of vagal reactivity, in which an amended Three Rs paradigm will be implemented for compatibility with the dynamic resilience framework. Hence, two research questions have driven the efforts of the current paper.

1. What vagal reactivity differences, if any, from the vmHRV parameters (rMSSD, pNN50, HF-HRV) emerge in the dynamic resilience level of individuals undertaking a dynamic decision-making task?
2. What temporal characteristics of task stress are the vmHRV reactivity parameters (rMSSD, pNN50, HF-HRV) sensitive to between the dynamic resilience groups for an amended Three Rs paradigm of a dynamic decision-making task?

Together, it is hypothesized that higher dynamic resilience groups will exhibit smaller vagal reactivity (i.e., small decreases in vmHRV reactivity scores), derived from vmHRV parameters (rMSSD, pNN50, HF-HRV), than lower dynamic resilience groups. Furthermore, it is hypothesized that higher dynamic resilience groups will exhibit smaller vmHRV reactivity during reactivity periods, and higher vagal stability (i.e., increases in vmHRV reactivity scores) following reactivity periods compared to lower dynamic resilience groups (Laborde et al., 2018).

## 2. Materials and Methods

### 2.1. Participants

Human ethics approval for the current study was granted by the Human research ethics committee, Faculty of Science and Built Environment, Deakin University, Australia. Participants were recruited via soft (e.g., social media) and hard (e.g., paper-flyers) copy advertisement. Potential participants were notified of the exclusion criteria, such as age restrictions (18–60), right hand dominant (to reduce systematic errors), being physically healthy (e.g., no known cardiovascular disease), and no psychopathological disorders; for example, (e.g., post-traumatic stress disorder) (Walker et al., 2019). In total, 60 eligible right-handed participants (37 male and 23 female), aged between 20 and 58 years (*M* = 30.57, *S.D* = 9.25), were recruited to participate in the current experiment. Following assessment of data quality, 14 participants were removed from the aggregated analyses. This concluded a total of 46 participants (31 males and 15 females), aged between 20 and 58 (*M* = 30.61, *S.D* = 9.51) for the analyses.

### 2.2. The Multi-Attribute Task Battery II (MATB-II)

**Figure 1:**
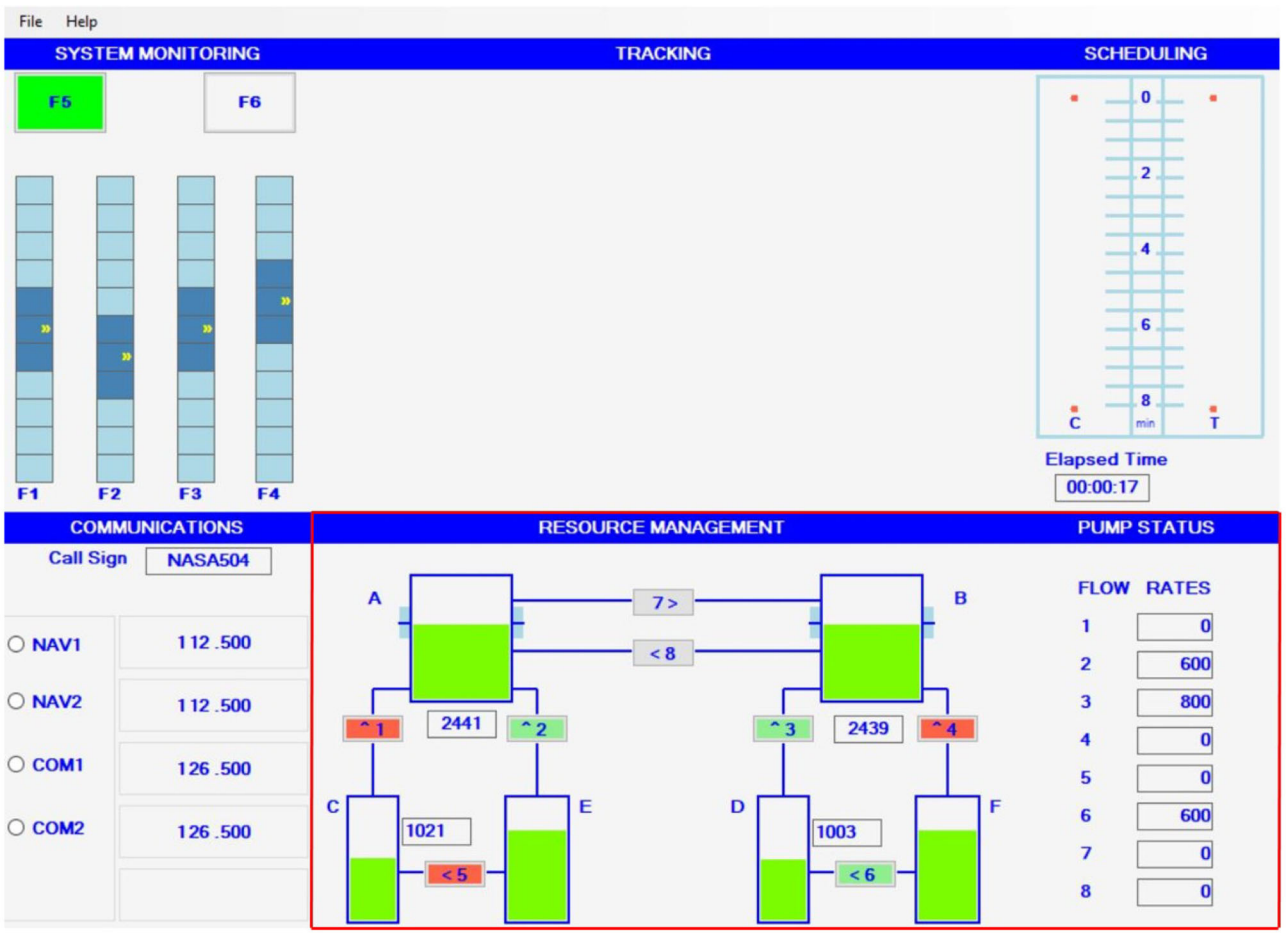
MATB-II user interface. RESMAN task located within red boundaries.

The MATB-II, depicted in Figure 1, was used as the task environment in the experiment (Santiago-Espada et al., 2011). The MATB-II is a computer-based task, popularized within the human factors research community, to assess human cognitive performance (Kennedy and Parker, 2017). The task interface presents four subtasks that target different cognitive functions of the user (e,g., sustained attention, executive functioning, auditory processing) (Santiago-Espada et al., 2011). The subtask interfaces were designed to present an aviation-familiar setting purposed with inducing stress within pilots and crew members within aviation sectors (Kennedy and Parker, 2017; Nixon and Charles, 2017). However, its applicability for other complex working domains has been supported, as the frequent, uncontrollable and unpredictable stressors imposed throughout tasks elicit similar responses across various domains (Kennedy and Parker, 2017). Furthermore, the subtasks are simple to learn and require short training periods; with task load and complexity adjustable through manipulations of temporal and spatial characteristics (Mortazavi et al., 2019). The MATB-II also facilitates flexible programming that allows for the subtasks to be presented simultaneously, alternatively, or singularly (Santiago-Espada et al., 2011).

The MATB-II's resource management (RESMAN) subtask was implemented to measure the dynamic resilience of users' executive functioning during a dynamic task. The RESMAN subtask was selected as it offers a dynamic, complex, and unpredictable task environment that targets the dynamic decision-making functions of one's executive functioning through requirements of strategizing, scheduling and executing actions (Buehler, 2018). This is achieved by presenting a generalized fuel management task in which individuals must manage the numeric “fuel” levels of two primary tanks. During the task, the primary tanks consume fuel leading to depleted fuel tanks. Users overcome depleting fuel tanks by transferring fuel from secondary tanks to the primary tanks via connecting pumps. However, over the course of the task, connecting pumps may fail and become temporally disabled, and preventing fuel transfer between tanks. In these situations, the user must then strategize the best way to overcome pump failures and redirect fuel to other tanks to counteract depletion in the primary tanks' fuel level. Lastly, the RESMAN subtask's performance output is formatted as a time series, thereby offering compatibility with the dynamic resilience framework.

As depicted in Figure 1, the task's interface presents six tanks connected by eight pumps. Each tank has specific roles and parameters within the task. Tanks “A” and “B” are primary tanks, in which individuals must manage to ensure that the fuel level is always as close to 2,500 units as possible. Tanks “C” and “D” are the finite supply tanks that have connecting pumps to supply additional fuel to the primary tanks. However, as tanks “C” and “D” only have a finite amount of “fuel,” the tanks are susceptible to becoming empty if mismanaged; leading to insufficient “fuel” levels to replenish fuel level depletions in the primary tanks. Lastly, tanks “E” and “F” are secondary tanks that have infinite quantities of fuel. Tanks “E” and “F” have pumps connecting to both the primary tanks and finite secondary tanks, which can be activated to replenish fuel depletion in fuel-needy tanks. Fuel is represented as the green component in the tanks and is further expressed via the numeric value overlaid across the tank graphics. All pumps have a one-directional fuel transfer as each pump have varied units of flow rate that determines how quick the “fuel” can be transferred between tanks. Flow rates are predetermined, in which the individual operator cannot alter.

### 2.3. Task Manipulations

The task manipulations of the phase conditions were identical to those presented by Crameri et al. (2020b). Together, these task manipulations were designed to acquire data suitable for dynamic resilience evaluation. Brief details of the task's temporal properties and difficulty manipulations are presented as follows.

**Figure 2:**
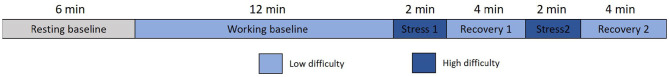
Experimental design detailing the temporal properties and task difficulty of the six phase conditions.

As depicted in Figure 2, each 30 min experimental trial consisted of six phase conditions, which imposed different task difficulty on the participants. This comprised:

- 6-min resting baseline (no task)
- 12-min working baseline (low task difficulty)
- 2-min stress period 1 (high task difficulty)
- 4-min recovery-to-working period 1 (low task difficulty)
- 2-min stress period 2 (high task difficulty)
- 4-min recovery-to-working period 2 (low task difficulty).

Two task difficulty levels were imposed within the experimental trial. Low level task difficulty, designed by Wang et al. (2017) MATB-II RESMAN level 3 configuration, was implemented to impose 3 simultaneous pump failures for 160 s of fail time over five minutes. High level task difficulty was configured upon Dynamic Adaptability Theory (Hancock and Warm, 1989), in which the failure of four simultaneous pumps occurred and alternated between various combinations of pumps failures every five to nine seconds.

The temporal sequence of the phases was designed to simulate a dynamic working environment in which the user would experience recurrent stress-inducing events. In accord with dynamic resilience theory, the history of task performance plays a prominent role in moderating the status of one's dynamic resilience (Hill et al., 2018b). Hence, the intention for the working baseline was to provide a period to build a history of task performance and induce a standardized mental set across participants (Walker et al., 2019). The high task difficulty stress periods were purposed with evoking experiences of task stress and failures for participants to overcome. Meanwhile, the low task difficulty recovery-to-work periods were designed to reduce task difficulty to working baseline conditions and evaluate how participants recovered and continued performing following stress periods.

### 2.4. Amended Three Rs Paradigm

An amended Three Rs paradigm was implemented toward compatibility with the dynamic resilience framework. Laborde et al. (2017)'s Three Rs paradigm was amended two-fold, (1) redefine rest and recovery phase parameters, and (2) the repetition of the Three Rs sequence. Firstly, the rest and recovery phase parameters were redefined to reflect how individuals' respond and recover to task stressors while working within an on-going task. This was achieved by applying a common workload (i.e., low difficulty) between the two phases. Furthermore, to better represent this paradigm for temporal, multi-event experimental designs, we retitled “rest” to “readiness” to reflect one's willingness to perform and preparedness to experience potential stressors. Secondly, the repetition of the sequence (i.e., Readiness-Reactivity-Recovery-Readiness-Reactivity-Recovery) provides an avenue toward psychophysiological assessments of how vmHRV reactivity interacts throughout the history of the task. The amended Three Rs paradigm overlaid the experimental design and set the following segments to acquire vmHRV:

- 2 min pre-task (no task) taken from minutes 2–4 of the resting baseline
- 2 min readiness period 1 (low task difficulty) taken from minutes 10–12 of the working baseline
- 2 min reactivity period 1 (high task difficulty) taken from minutes 12–14 of stress period 1
- 2 min recovery period 1 (low task difficulty) taken from minutes 14–16 of recovery-to-working period 1
- 2 min readiness period 2 (low task difficulty) taken from minutes 16–18 of recovery-to-working period 1
- 2 min reactivity period 2 (high task difficulty) taken from minutes 18–20 of stress period 2
- 2 min recovery period 2 (low task difficulty) taken from minutes 20–22 of recovery-to-working period 2.

Accordingly, this produced seven, two-minutes segments for each vmHRV parameter. While traditional vmHRV guidelines suggest five-minute minimum vmHRV segments, shorter recordings of 60 s have been validated (Malik et al., 1996; Smith et al., 2013). Therefore, the two-minute segments of vmHRV parameters were used to calculate vmHRV reactivity scores.

### 2.5. EQ02 Lifemonitor

The EQ02 LifeMonitor (Equivital, Cambridgeshire, England) was used as the hardware device to capture individuals' ECG data. The EQ02 lifemonitor is accurate and validated tool for ECG and HRV research, albeit its proneness for higher artifact load (Akintola et al., 2016). The EQ02 Lifemonitor consists of a sensor electronics module (SEM) and a sensor belt. The sensor belt presents as a vest that comprises two sensors that fit across the midsection of the user's torso. The SEM acquires data at 256 Hz. To export the acquired logged data, the SEM is connected to a PC via remote USB connectivity and using its software, Equivital Manager (Version 2.5.3.130, Equivital, Cambridgeshire, England), the logged data can be exported and formatted into comma separated values (csv) files for further analysis.

### 2.6. Experimental Protocol

Upon arrival, eligible voluntary participants were provided with a plain language statement and consent forms to be signed to provide consent to participate. A demographics was presented to participants to complete. Once completed, MATB-II RESMAN instruction manuals were provided to participants, detailing the task's operation instructions and objectives. As the simplicity of the MATB-II RESMAN task does not require long training periods, approximately 10 min training period was allocated for participants to familiarize themselves with the RESMAN task. During the training period, participants were monitored for competency and encouraged to ask questions if unclear on the task instructions and mechanics. Upon the conclusion of the training period, participants were fitted with physiological measuring equipment. This included the Equivital vest; and also an electroencephalography (EEG) and finger-based galvanic skin response (GSR) sensor, and headphones that produced an acoustic startle for research not presented in this paper (see Crameri et al., 2020a for the findings on electrodermal activity and dynamic resilience). Once fitted with the physiological measurement equipment, a 6 min resting baseline was conducted. During the resting baseline, participants were directed to sit and relax with their eyes open and to avoid talking and moving. After the physiological parameters were acquired during the resting baseline, participants commenced the 24 min experimental trial. Upon finishing the trial, participants were assisted in removing the physiological measurement equipment, had any further questions about the experiment answered, and directed to leave the experiment area.

### 2.7. Dynamic Resilience Groups-Based on Performance Quantification

Participants' dynamic resilience was derived from the RESMAN subtask's performance via (Crameri et al., 2020b)'s proposed measure. Task performance was calculated by converting the two primary tanks' fuel units (*tank*_*i*_) into the total percentage error from the optimal fuel level,

(1) $$\begin{array}{r} E_{i}\left(t\right)=abs\left(x_{i}\left(t\right)-Target_{i}\right)/Target_{i}\times 100 \end{array}$$

where *E*_*i*_(*t*) is the % error of *tank*_*i*_ at time point *t*. *x*_*i*_*(t)* is the fuel level of *tank*_*i*_ at time *t*. *Target*_*i* = 1, 2_ = 2,500, is the optimal fuel level, and *abs* is the absolute value function. The overall performance errors at each time point, *t, P*_*E*_*(t)*, was calculated via,

(2) $$\begin{array}{r} P_{E}\left(t\right)=\frac{1}{n}\times \sum E_{i}\left(t\right) \end{array}$$

where *n* is the number of tanks, and lower *P*_*E*_(*t*) values reflect less error and, therefore, higher performance. Crameri et al. (2020b)'s dynamic resilience equation was calculated by firstly conducting a first-order autoregressive [AR(1)] modeling on the time points of the performance metric *P*_*E*_(*t*). The performance was recorded at each 15 s interval. A window length of 10-points (150 s) was used, with the window moving 1-point (15 s) at each iteration.

Dynamic resilience scores for the *kth* stress-recovery period ($R_{AR}^{k}$) was derived as,

(3) $$\begin{array}{r} R_{AR}^{k}=SP_{AR}^{k}-RP_{AR}^{k} \end{array}$$

Where $SP_{AR}^{k}$ is the maximum AR(1) value during the *k*^*th*^ stress period, and $RP_{AR}^{k}$ is the minimum AR(1) value during the *kth* recovery period. For the current study, *k* = 1, 2, thus, participants produce two dynamic resilience values for each stress-recovery block, with higher scores reflecting higher dynamic resilience. To classify participants into dynamic resilience groups (e.g., lower, moderate, higher), the participants' $R_{AR}^{1}$ and $R_{AR}^{2}$ scores were paired and evaluated via a K-means cluster analysis (Crameri et al., 2020b).

### 2.8. vmHRV Processing

Raw electrocardiogram (ECG) data was exported from the Equivital into csv data files, and processed in MATLAB. Raw ECG data for each participant was inspected for signal quality and those deemed faulty or extremely noisy were excluded from further analyses. The data was then segmented into respective experimental conditions shown in Figure 2. For the working baseline, data recorded between the 4th and 8th min were only considered, as this would likely reduce the likelihood of practice and boredom effects in the data. For each segment the following processing pipeline was adopted.

The QRS complexes of the ECG recording were detected using the Pan-Tompkins QRS detection algorithm (Pan and Tompkins, 1985). The RR intervals were calculated by taking the time differences between two successive R-peaks. The derived RR-series comprising of the RR intervals was examined and corrected for any missed and/or extra beats using a quotient filter (Bartels et al., 2017; Hettiarachchi et al., 2019). This yielded the inter-beat-interval (IBI) series which is used for the vmHRV metric calculation in millisecond (ms) units.

The vmHRV data used in this study was calculated following the guidelines given in the Task force of the European society of cardiology and the North American society of pacing and electrophysiology (Malik et al., 1996; Berntson et al., 1997), using the time domain method and the frequency domain methods. Prior to frequency domain analysis, the IBI series was interpolated and then re-sampled using a sampling frequency of 4 Hz (Bartels et al., 2017) and a linear detrend was applied using the mean. The HF-HRV frequency domain parameter was evaluated using the power spectral density, which was calculated by using a Fast Fourier Transformation in this study. HF-HRV comprises the total spectral power of all RR intervals between 0.15 and 0.4 Hz. Meanwhile, the time domain method analyses the IBIs and capture the variation in time occurring between the beats. The time domain vmHRV parameters included in the study comprise the rMSSD and the pNN50.

vmHRV reactivity scores were calculated between each successive vmHRV segment. vmHRV reactivity was calculated via residualized change scores between each successive vmHRV segment as a means of controlling for individual difference in baseline vmHRV (Manuck et al., 1989; Howard et al., 2017). This approach toward measuring vmHRV stress reactivity is applied to overcome issues regarding the law of initial values phenomenon (Larkin, 2006). In the context of physiological responses, this phenomenon details how differences in resting levels of physiological systems will influence the magnitude of an emotional stimulus to the respective physiological system under review. For example, statistically less vmHRV reactivity may be exhibited in individuals possessing lower resting vmHRV between task conditions than individuals with lower vmHRV due to physiological ceiling effects that limit significant physiological changes (Larkin, 2006; Diller et al., 2011). Hence, postulating that resting vmHRV is a predictor of vmHRV reactivity and will affect results if it is not controlled for. To remove this effect, the residualized change scores are calculated by regressing a vmHRV segment on the successive vmHRV segment (e.g., readiness period 1 regressed on reactivity period 1) (Diller et al., 2011). Ultimately, producing six segments of vmHRV reactivity scores, in which negative scores reflect elevated vagal reactivity and positive scores reflect vagal stability.

### 2.9. Statistical Analysis

Repeated measures mixed analysis of variance (ANOVA) were used to analyse the effects of within subject factor experimental condition (six segments) and between subject factor resilience groups (three groups: Lower, Moderate, and Higher) on the time domain and frequency domain HRV parameters. The assumptions for parametric testing were evaluated prior to each analysis. Outliers were identified via the inspection of the studentized residuals of each HRV metric. Outliers were identified as those that were outside the boundaries of ±3, to which one outlier was located and removed.

Shapiro-Wilks tests of normality was conducted on for each HRV metric across each condition by each group. A natural log transformation was applied to HF-HRV (denoted lnHF-HRV), rMSSD (denoted lnrMSSD), and pNN50 (denoted lnpNN50) data sets to reduce skewness prior to calculating residualized change scores. A Levene's test of homogeneity of variance revealed equal variance for lnHF across all HRV metrics of dynamic resilience groups across conditions (*p* < 0.05). Homogeneity of covariance was supported by Box's test of equality of covariance matrices (*p* > 0.05) for lnHF-HRV but not lnrMSSD (*p* < 0.019) and lnpNN50 (*p* = 0.001); however, as homogeneity of variance was not violated for these HRV metrics violations of this assumption was relaxed. Lastly, Mauchly's test of sphericity was inspected for violations in sphericity. A Greenhouse-Geisser correction was applied when violations were verified (*p* < 0.05).

## 3. Results

### 3.1. Dynamic Resilience Groups

**Figure 3:**
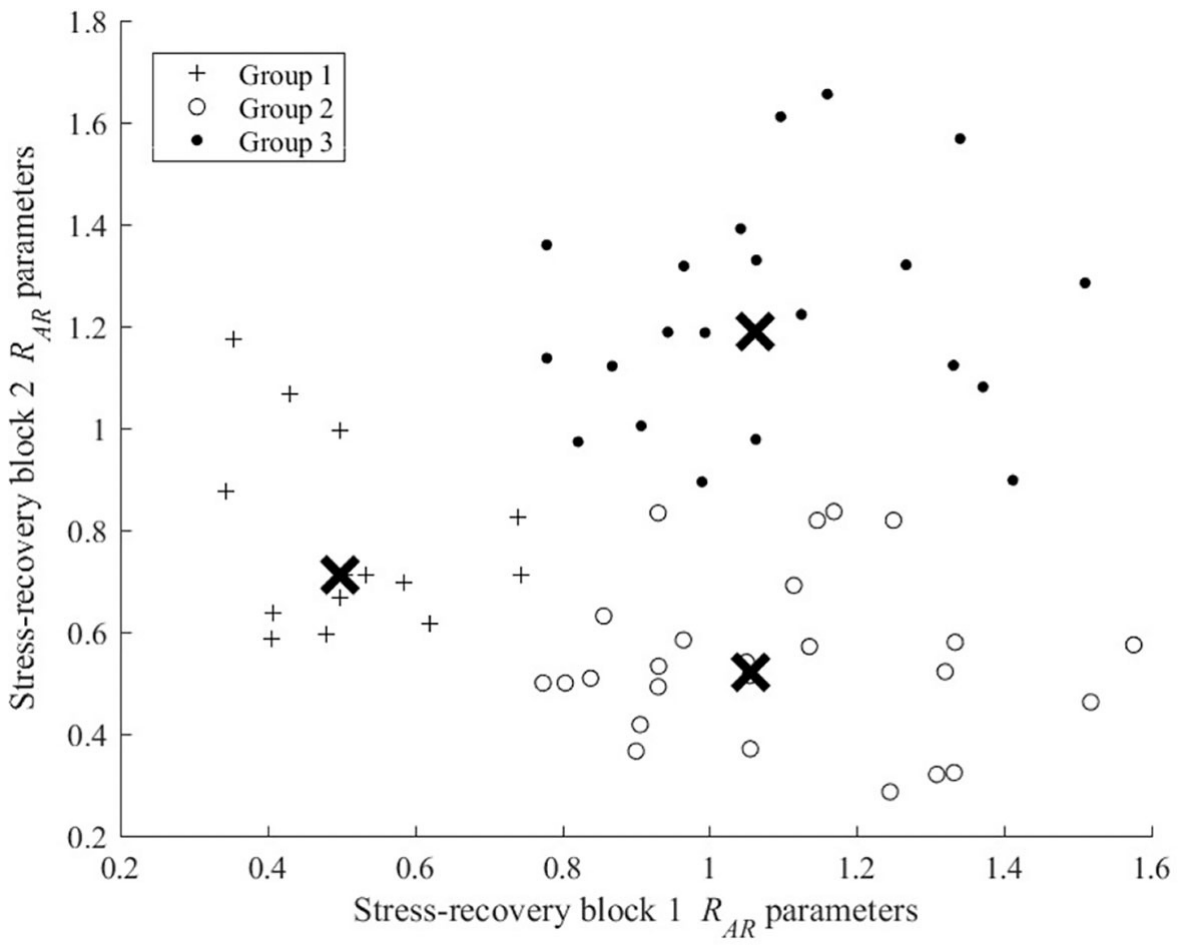
Dynamic resilience groups formed via K-means three clustering of $R_{AR}^{k}$ (*k* = 1, 2) scores. (**X** denotes group centroid).

To classify participants into different dynamic resilience groups, a K-means cluster analysis was conducted. Participants' $R_{AR}^{1}$ and $R_{AR}^{2}$ scores were paired for analysis. As depicted in Figure 3, the analysis revealed 3 distinguishable groups. Clusters for Group 1 produced the smallest group (*n* = 14), with participants' ages ranging between 26 and 58 (*M* = 35.50, *S*.*D* = 11.40), with 6 males and 8 females. Group 1's $R_{AR}^{1}$ scores clustered between 0.34 and 0.74 and between 0.58 and 1.17 in $R_{AR}^{2}$. Twenty-five participants (17 males and 8 females), aged between 20 and 56 (*M* = 29.88, *S*.*D* = 8.53) were clustered into Group 2. Group 2's $R_{AR}^{1}$ scores and $R_{AR}^{2}$ ranged between 0.77 and 1.58, and 0.29 and 0.84, respectively. Lastly, Group 3 comprised 21 participants (14 males and 7 females), aged between 20 and 33 (*M* = 28.10, *S*.*D* = 7.50). $R_{AR}^{1}$ and $R_{AR}^{2}$ ranged between 0.78 and 1.51, and 0.99 and 1.66, respectively.

In evaluating the $R_{AR}^{1}$ and $R_{AR}^{2}$ scores for the different groups, it appears that participants were clustered into groups based upon their dynamic resilience decay over the trial. More specifically, participants in Group 1 appears to be those that possessed the lowest dynamic resilience over the trial, with decays in dynamic resilience occurring during stress-recovery block 1. Participants in Group 2 appear to represent those who exhibited higher dynamic resilience during stress-recovery block 1, yet exhibited dynamic resilience decaying in stress-recovery block 2; thereby, in the context of the groups could be considered those that possessed moderate dynamic resilience for the task. Lastly, Group 3's participants appear to represent those that possessed higher dynamic resilience throughout the trial, as these participants exhibited relatively higher dynamic resilience during both stress block 1 and stress block 2.

Given the distinct groups created by the cluster analysis, participants were analyzed in the context of their respective group. Following the omission of participants with noisy ECG signals and the identified outlier, the lower dynamic resilience group comprised nine participants; moderate dynamic resilience group included 18 participants; and the higher dynamic resilience group comprised 18 participants. As recommended by Guo et al. (2013), a specialized repeated measures statistical power software, GLIMMPSE (https://glimmpse.samplesizeshop.org/), was employed to ensure sufficient statistical power was present for the following analyses. The software tool calculated a statistical power of 0.960, thereby enhancing confidence toward the mitigation of Type II errors.

### 3.2. vmHRV Reactivity Between Dynamic Resilience Groups

**Figure 4:**
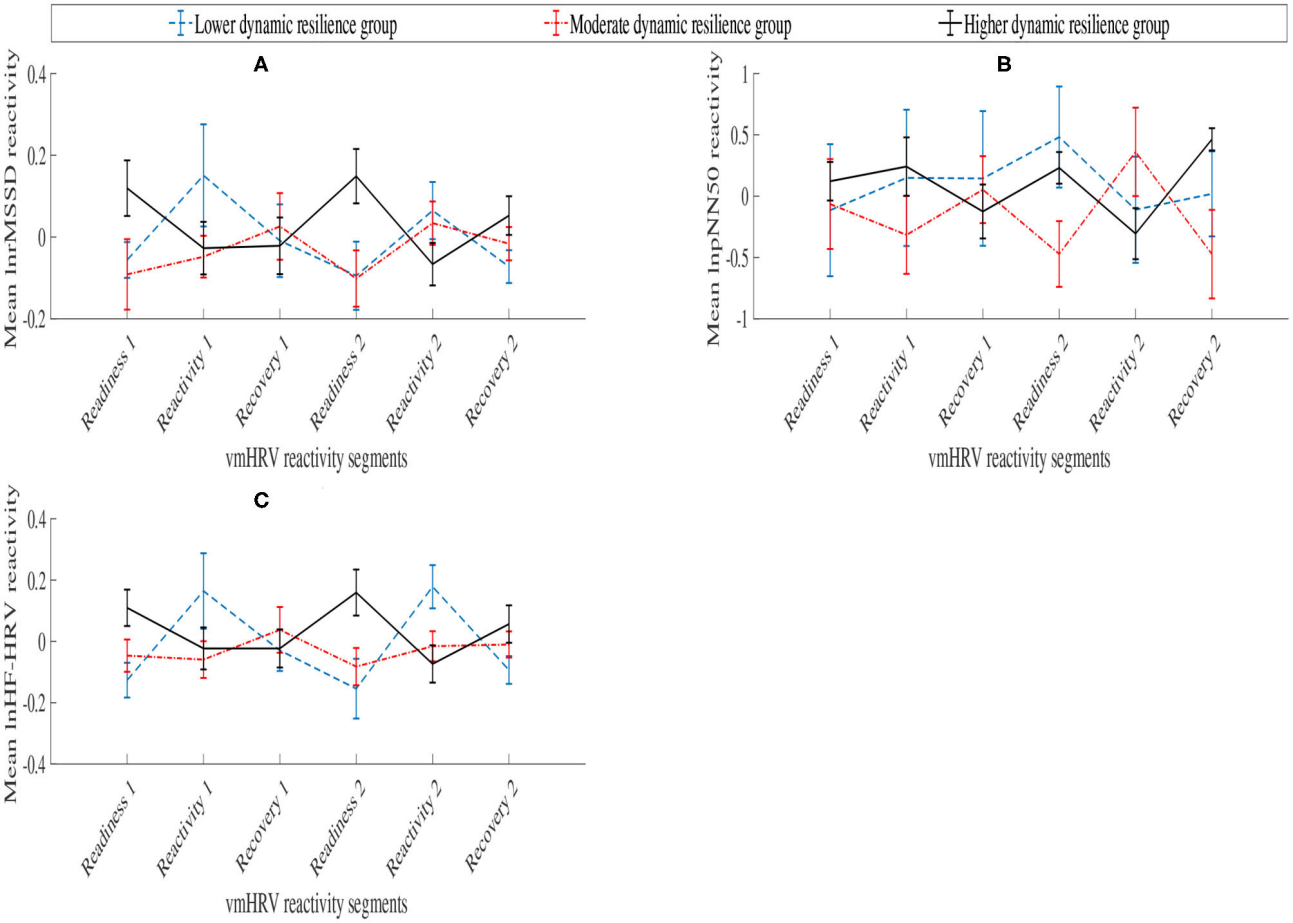
Aggregated vmHRV reactivity scores between dynamic resilience group across vmHRV reactivity segments. **(A)** lnrMSSD; **(B)** lnpNN50; **(C)** lnHF-HRV.

Table 1 and Figure 4 presents the variation of the vmHRV reactivity score of each parameters, lnrMSSD, lnpNN50, and lnHF-HRV between each dynamic resilience group across the six HRV reactivity segments.

**Table 1:** Mean (SD) values of lnrMSSD, lnpNN50, lnHF for each vmHRV segment across the three dynamic resilience groups [Low (*n* = 9), Medium (*N* = 18), and High (*n* = 18)].

**Group**	**Segment**					
	**Readiness 1**	**Reactivity 1**	**Recovery 1**	**Readiness 2**	**Reactivity 2**	**Recovery 2**
**lnrMSSD**						
Lower	−0.056	0.151	−0.009	−0.095	0.064	−0.072
	(*0.131*)	(*0.375*)	(*0.267*)	(*0.250*)	(*0.210*)	(*0.120*)
Moderate	−0.091	−0.048	0.026	−0.102	0.034	−0.016
	(*0.365*)	(*0.217*)	(*0.346*)	(*0.292*)	(*0.225*)	(*0.173*)
Higher	0.119	−0.027	−0.021	0.149	−0.066	0.053
	(*0.288*)	(*0.273*)	(*0.294*)	(*0.282*)	(*0.221*)	(*0.201*)
**lnpNN50**						
Lower	−0.115	0.149	0.144	0.482	−0.111	0.019
	(*1.615*)	(*1.667*)	(*1.648*)	(*1.237*)	(*1.300*)	(*1.041*)
Moderate	−0.065	−0.3167	0.053	−0.472	0.361	−0.473
	(*1.555*)	(*1.349*)	(*1.158*)	(*1.137*)	(*1.530*)	(*1.531*)
Higher	0.122	0.242	−0.125	0.231	−0.306	0.464
	(*0.667*)	(*1.009*)	(*0.934*)	(*0.548*)	(*0.884*)	(*0.385*)
**lnHF**						
Lower	−0.126	0.164	−0.029	−0.154	0.178	−0.093
	(*0.170*)	(*0.369*)	(*0.201*)	(*0.292*)	(*0.212*)	(*0.136*)
Moderate	−0.046	−0.059	0.038	−0.082	−0.016	−0.010
	(*0.224*)	(*0.254*)	(*0.316*)	(*0.258*)	(*0.207*)	(*0.181*)
Higher	0.110	−0.023	−0.023	0.159	−0.073	0.057
	(*0.252*)	(*0.291*)	(*0.264*)	(*0.318*)	(*0.258*)	(*0.259*)

A 3 (Dynamic resilience group [Lower dynamic resilience group, Moderate dynamic resilience group, Higher dynamic resilience group]) x 6 (vmHRV reactivity segment [Readiness1, Reactivity1, Recovery1, Readiness2, Reactivity2, Recovery2]) mixed ANOVA was conducted on lnrMSSD reactivity scores, lnpNN50 reactivity scores, and lnHF-HRV reactivity scores. No statistically significant interaction was supported between dynamic resilience group and vmHRV reactivity segment for lnrMSSD reactivity scores [$F_{\left(7.611,159.840\right)}=1.773,p=0.09,\eta^{2}=0.078$]. A main effect for dynamic resilience group was supported [$F_{\left(2,42\right)}=3.562,p=0.037,\eta^{2}=0.145$]; yet, a main effect for vmHRV reactivity segment was not supported [$F_{\left(3.806,159.840\right)}=0.128,p=0.986,\eta^{2}=0.003$]. A *post-hoc* analysis with Bonferroni correction revealed that the higher dynamic resilience group exhibited statistically significantly smaller lnrMSSD reactivity scores across the vmHRV reactivity segments than the moderate dynamic resilience group (*M*=-0.067, *p* = 0.011).

No statistically significant interaction was supported between dynamic resilience group and vmHRV reactivity segment for lnpNN50 reactivity [$F_{\left(6.011,126.226\right)}=1.506,p=0.181,\eta^{2}=0.067$]. Additionally, no main effects for dynamic resilience groups [$F_{\left(2,42\right)}=0.950,p=0.395,\eta^{2}=0.043$] and vmHRV reactivity segments [$F_{\left(3.005,126.226\right)}=0.044,p=0.988,\eta^{2}=0.001$] were supported.

A statistically significant interaction was supported between dynamic resilience group and vmHRV reactivity segment on lnHF-HRV reactivity [$F_{\left(7.205,151.300\right)}=2.589,p=0.014,\eta^{2}=0.110$]. Simple main effects for dynamic resilience groups across each phase, of which statistical differences were supported in Readiness 1 [$F_{\left(2,42\right)}=3.881,p=0.028,\eta^{2}=0.156$] and Readiness 2 [$F_{\left(2,42\right)}=4.709,p=0.014,\eta^{2}=0.183$]. *Post-hoc* analyses with Bonferroni correction revealed statistically significant lnHF-HRV reactivity between the lower dynamic resilience group and the higher dynamic resilience group in Readiness 1 (*p* = 0.046) and Readiness 2 (*p* = 0.034). Meanwhile, *post hoc* analyses with Bonferroni correction revealed statistically significant lnHF-HRV reactivity between the moderate dynamic resilience group and the higher dynamic resilience group in Readiness 2 (*p* = 0.049). No simple main effects were supported for vmHRV reactivity segments across groups (*p* > 0.05).

## 4. Discussion

The current study's objective was to examine if distinct vagal reactivity emerges based upon dynamic resilience classification within an on-going, dynamic decision-making task. Additionally, the current study also sought to examine if an amended Three Rs paradigm offered value in evaluating vagal reactivity across temporal characteristics of a dynamic decision-making task between dynamic resilience groups. Firstly, this was achieved by measuring and classifying participants' dynamic resilience via a dynamic decision-making task that presented two stress and recovery-to-working periods (Hill et al., 2018a; Crameri et al., 2020b). Classification of participants' dynamic resilience revealed three dynamic resilience groups that appeared to be representative of the stage in which participants' dynamic resilience diminished (i.e., during stress block 1, stress block 2, or not at all) (Crameri et al., 2020b). Cardiac vagal control was derived through three vmHRV parameters that comprised, rMSSD, pNN50, and HF-HRV (Malik et al., 1996; Berntson et al., 1997; Laborde et al., 2017). To assess vagal reactivity between task conditions and promote compatibility within the dynamic resilience framework, vmHRV segments were created through an amended Three Rs paradigm. This paradigm overlayed the dynamic resilience paradigm and produced seven vmHRV segments to which six vmHRV reactivity score were calculated. An aggregated analysis of each groups' vmHRV reactivity score across each vmHRV parameter was conducted to identify if significant interactions or differences existed between the groups and the vmHRV reactivity segments. It was hypothesized that higher dynamic resilience groups would exhibit smaller vagal reactivity (i.e., small decreases in vmHRV reactivity scores), derived from vmHRV parameters (rMSSD, pNN50, HF-HRV), than lower dynamic resilience groups. Furthermore, it was hypothesized that higher dynamic resilience groups would exhibit smaller vmHRV reactivity during reactivity periods, and higher vagal stability (i.e., increases in vmHRV reactivity scores) following reactivity periods compared to lower dynamic resilience groups (Laborde et al., 2018). Overall, the findings partially supported the hypotheses as statistically significant differences were supported in lnrMSSD and lnHF-HRV reactivity scores but not lnpNN50 reactivity scores between dynamic resilience groups; and the higher dynamic resilience group exhibited statistically higher vagal stability than the moderate dynamic resilience group following the reactivity period.

Prior to interpreting the results, the context in which vmHRV reactivity scores are assessed is important; as different interpretations may be present for similar patterns of vmHRV reactivity scores. For example, while larger negative vmHRV reactivity scores is widely accepted as a physiological adaptation of self-regulation toward threatening and challenging tasks (Hjortskov et al., 2004; Seery, 2011; Laborde et al., 2017, 2018), reasons for positive vmHRV reactivity scores are two-fold. Firstly, positive vmHRV reactivity scores could indicate the individual has overcome task stress and has returned to a comfortable working state (Laborde et al., 2018). Conversely, positive vmHRV reactivity scores may be a response to “giving up” on the task due to difficulty. Depending on the nature of the task and the ramifications that comes with yielding, individuals may vary in their physiological adaptive response. If conceding from a task that had high ramification for failure, individuals may become overstimulated from stressful task events due to contributing somatic anxiety symptoms in which large decrease in vmHRV reactivity scores are exhibited (Taylor et al., 2009; Thompson et al., 2015). Alternately, if there are no consequences for “giving up” (e.g., participating in a laboratory study), vmHRV reactivity scores may exhibit increases in which the stressful task events no longer elicit an effect as the individual has no emotional investment in the outcome.

Supporting their close parametric relationship, similar patterns of lnrMSSD and lnHF-HRV reactivity scores were exhibited between dynamic resilience groups across the vmHRV reactivity segments. In the context of dynamic resilience during the first stress block, vmHRV reactivity scores during reactivity 1 and recovery 1 revealed elevated vagal reactivity in the higher dynamic resilience group. Vagal reactivity during these vmHRV reactivity segments were relatively small and may reflect positive self-regulation of higher workload during the stress period, and self-regulatory recovery processes during recovery 1 (Laborde et al., 2018). Meanwhile, despite recording higher performance, the moderate dynamic resilience group exhibited high vagal reactivity during the reactivity 1 vmHRV segment. This may signal a large investment of self-regulatory resources toward overcoming the more challenging task event, that may have been a contributor to the performance decline in the following stress block. However, during recovery 1, the moderate dynamic resilience group exhibited vagal stability, potentially indicating an initial relief to the reduced workload. The lower dynamic resilience group appeared to relax during this stress period, as their vmHRV parameter reactivity score increased. Given the context of this trend in which the lower dynamic resilience group exhibited poorer performance during stress period 1, this vmHRV reactivity pattern was possibly due to task disengagement or withdrawal of emotional investment as a consequence of the task difficulty being too high. However, during the recovery 1, the lower dynamic resilience group exhibited small elevated vagal reactivity that may suggest their re-engagement into the task.

During the second stress block, large vmHRV reactivity score differences emerged between higher and moderate dynamic resilience groups. In particular, the moderate dynamic resilience group exhibited high vagal reactivity, whereas the higher dynamic resilience group appeared to exhibit vagal stability in which vmHRV reactivity scores dramatically increased. In the context of the respective groups' performance, the moderate dynamic resilience group's pattern of vagal reactivity may reveal indications of decaying self-regulatory processes that have arisen due to the residual effects of previous stressful task events (Laborde et al., 2018). Conversely, the higher dynamic resilience group appears to exhibit vagal stability in which they may have habituated to the task difficulty and no longer require the physiological adaptations to successfully undertake the task (Lü et al., 2016; Howard et al., 2017). lnrMSSD and lnHF-HRV reactivity scores during reactivity 2 revealed different vagal outcomes for the moderate dynamic resilience group. lnrMSSD reactivity scores were positive, thereby suggesting that the moderate dynamic resilience group experienced vagal stability; as opposed to small negative lnHF-HRV reactivity scores. Nevertheless, this may indicate levels of disengagement or withdrawal of emotional investment from the task due to the increased workload in which the moderate dynamic resilience group can no longer cope with.

Meanwhile, the higher dynamic resilience group exhibited high vagal reactivity during the reactivity 2. While appearing to habituate to the task difficulty during the readiness 2 vmHRV reactivity segment, the higher dynamic resilience group did not exhibit habituating effects during the second stress block as there was larger elevated vagal reactivity in reactivity 2 than reactivity 1. This may highlight the temporal effects of an on-going task in which the role of task history and current task status (i.e., performance level) influences habituation. Following reactivity 2, the higher dynamic resilience group vagally stabilized during recovery 2 indicating relief from lower task load. Finally, the lower dynamic resilience group exhibited a similar vmHRV reactivity pattern to the first stress block, in which vagal reactivity was present during readiness 2 and recovery 2, and vagally stabilizing during reactivity 2. Again, this is likely due to disengagement with the task during higher task load, and re-engagement during lower task load.

Statistically, analyses partially supported the hypothesis that the higher dynamic resilience groups would exhibit smaller vagal reactivity than the lower dynamic resilience groups; as lnrMSSD reactivity scores were overall significantly lower in the higher dynamic resilience group when compared to the moderate dynamic resilience group across vmHRV reactivity segments. According to the NVI model, this may suggest that higher dynamic resilience may be a product of enhanced temporal processing of the CAN (Thayer et al., 2009; Smith et al., 2020). In particular, dynamic resilience within a dynamic decision-making task may index the efficiency of temporal executive functions, in which the individual will possess superior strategizing and scheduling toward executing optimal courses of action. Additionally, dynamic resilience may also provide an index of temporal characteristics of emotional-regulation that facilitate the regulation of stressful or challenging events (Seery, 2011; Smith et al., 2017). However, neither higher or moderate dynamic resilience groups' vmHRV reactivity scores were statistically different from the lower dynamic resilience group; yet this was potentially due to task disengagement.

While analyses of lnrMSSD reactivity scores supported global difference between higher and moderate dynamic resilience groups, a significant interaction was supported in the analysis of lnHF-HRV reactivity scores between dynamic resilience groups across vmHRV reactivity segments. Further analyses of simple main effects revealed that higher dynamic resilience promoted vmHRV stability during readiness 2; thereby partially supporting the hypothesis that higher dynamic resilience groups would exhibit higher vagal stability following reactivity periods compared to lower dynamic resilience groups. This finding may further explain why the higher dynamic resilience group overcame the proceeding stress period as opposed to the vagally reactive moderate dynamic resilience group. Firstly, as aforementioned, higher dynamic resilience may have promoted habituation to task difficulty in which the individuals no longer exhibit vmHRV reactivity. According to an extended theory of the NVI model titled, the vagal tank theory (Laborde et al., 2018), inhibiting vmHRV reactivity may temporally enhance the higher dynamic resilient individuals' capacity of physiological resources that are called upon for self-regulation during stressful or challenging events. Hence, as the moderate dynamic resilience group did not habituate to the task during readiness 2, their vagal expenditure limited that availability of future physiological resources and, consequently, led to respective group members being overwhelmed by the task difficulty during reactivity 2.

As a point forward for operationalizing, the dynamic resilience concept and measure is still in its early infancy and requires a plethora of empirical research to validate its value across various human factors and human performance domains. Dynamic resilience was developed for human performance purposes, with the primary aim directed at predicting the on-set of irreversible performance declines (Hill et al., 2018a,b). The construct itself should not be conceived as a stable personal attribute like trait resilience, but a malleable cognitive process that may simultaneously change across various tasks and settings. However, the integrity of dynamic resilience cognitive framework is quite thin, as Hill et al. (2018b)'s proposed methodological avenues for dynamic resilience appear to be more reflective of task proficiency than cognitive processes. Clearly, task proficiency will contribute to the build of dynamic resilience as training and experience will mitigate the expenditure of cognitive and physical resources during task engagement, as well as when overcoming task stressors. As for cognitive contributors, the current study presents a potential avenue via the CAN. The significant link between vmHRV, cognitive performance and dynamic resilience classifications support a potential CAN-dynamic resilience interaction. The findings suggest that dynamic resilience may, at least partially, index the temporal processes of the CAN during executive functioning tasks. Hence, suggesting dynamic resilience may be built upon task proficiency and the efficiency of CAN functioning.

### 4.1. Methodological Considerations

The current experimental design aimed to impose recurring stress-recovery periods in the pursuit of measuring dynamic resilience. However, as observed in the current study, it was susceptible to participant disengagement with the task due to boredom or more likely task difficulty. This appears to be evident in the lower dynamic resilience group as their vmHRV reactivity patterns did not follow the trends of the other groups. Hence, experimental designs need to consider methods to enhance anxiety to task failures in order to more accurately capture physiological measures of lower dynamic resilience individuals or groups. This is one of the primary limitations of the task stress imposed in laboratory environments as it will never simulate the fidelity of task stress imposed in ecological task environments. This is largely due to the ramification for failure being predominantly absent in laboratory studies compared to the ramification for failure in operational task environments where personnel may be at risk of job security/selection, loss of equipment, and in extreme situations, loss of life. However, laboratory studies will always be of value given their relative low cost, flexibility in testing non-experts, and the capability to directly target cognitive functions of interest. Given this, potential considerations for laboratory studies to generate “buy in” from participants may be through competition, as lower HRV is found in competing athletes with higher cognitive and somatic anxiety psychometric scores (Fortes et al., 2017). Furthermore, teamwork may also generate more ecological physiological measurements as social desirability to succeed and not be seen as a weak link may increase “buy in” from participants and produce somatic anxiety responses during task failures. Ultimately, through human-interaction participants may be more inclined to commit to the experiment and reduce the gap between laboratory and ecological settings.

Further methodological consideration pertains to the number of confounding variables that must be accounted for when conducting vmHRV research (Laborde et al., 2017). In particular, during the experiment, participants wore physiological equipment and experienced acoustic startle stimuli for purposes not related to the current research (see Crameri et al., 2020a). There is a possibility that the additional physiological equipment and external factors contributed to task disengagements and dynamic resilience declines due to physical discomfort. Moreover, the acoustic startle stimuli are unlikely to affect vmHRV as the task effects are more likely to mask physiological startle responses (Walker et al., 2019). Furthermore, large quantities of demographics pertaining to participants' physiology and lifestyle factors are desired to provide adequate descriptions of the sample examined. While this current study did not capture a large battery of specific physiological and lifestyle factors, it did record age, sex, and asked required participants to be physically and psychologically healthy. Furthermore, the use of the residualized change scores calculation controlled for the individual differences between participants, thereby mitigating their internal effects (Diller et al., 2011; Howard et al., 2017). Nevertheless, specific cardiac screening measures should be considered in future research.

## 5. Future Research and Conclusion

To conclude, the finding support differences in vagal reactivity between dynamic resilience groups, albeit only in dynamic resilience groups hypothesized to be engaged throughout the task. It appears that higher dynamic resilience reflects more efficient functioning of the CAN, in which these individuals' exhibit less vmHRV reactivity during higher stress periods and greater vmHRV stability during recovery periods. This pattern of vmHRV activity may allow higher dynamic resilience individuals to conserve physiological resources toward prolonged coping within an on-going, complex task environment. Lastly, the significant interaction within the lnHF-HRV reactivity parameter partially supported the implementation of an amended Three Rs paradigm to evaluate if vagal reactivity can be temporally evaluated within a dynamic decision-making task between dynamic resilience groups.

According to the existing literature, this study provides a pilot effort toward pairing vmHRV to dynamic resilience. The empirical support of vmHRV and dynamic resilience offers a step forward as previous efforts to link physiological measures, such as skin conductance levels, to dynamic resilience were deemed insensitive (Crameri et al., 2020a). Hence, this research presents several avenues for future research to further ground the dynamic resilience theory.Given the empirical support linking trait resilience to self-regulation and dynamic resilience to temporal self-regulation and performance outcomes, these collective characteristics of resilience are likely to influence the neurobiological response to stress (Thayer et al., 2009; Laborde et al., 2018). Therefore, one path comprises examining if the convergence of trait resilience and dynamic resilience measures further distinguishes vmHRV and enhances the resilience profile toward greater markers of self-regulation and performance. A further research path could examine classifications and performance trajectory via machine learning methods. By testing combinations of performance and physiological measures for predictive power of dynamic resilience and performance, future dynamic resilience assessments can be enhanced and automated to provide quick accurate information of working personnel for industry organizations. Lastly, dynamic resilience theory and metrics could be examined as part of a more global cognitive performance theory and assessments such as Cognitive Readiness constructs (Grier, 2012; O'Neil et al., 2014; Crameri et al., 2019) or Cognitive Fitness assessments (Aidman, 2020). This would be most suitable at the operational and tactical levels of cognitive readiness, and either the cognitive gym, advanced cognitive training, or mission-ready training of the cognitive fitness framework depending on the training or monitoring objectives; for example, fatigue tolerance assessments (Aidman et al., 2018; Crameri et al., 2019; Aidman, 2020). Ultimately, through the aforementioned future research paths, dynamic resilience may become a valued component of operational performance predictions.

## Data Availability Statement

The raw data supporting the conclusions of this article will be made available by the authors, without undue reservation.

## Ethics Statement

The studies involving human participants were reviewed and approved by Faculty Human Ethics Advisory Group, Deakin University, Australia. The patients/participants provided their written informed consent to participate in this study.

## Author Contributions

All authors contributed to the conception and design of the study. LC conducted the experiment for data collection purposes, performed the statistical analyses, and wrote the first draft of the manuscript. IH wrote the algorithms for signal processing of the physiological data. All authors contributed to the manuscript revision, read and approved the submitted version.

## Conflict of Interest

The authors declare that the research was conducted in the absence of any commercial or financial relationships that could be construed as a potential conflict of interest.
